# 

**Published:** 1859-01

**Authors:** 


### THE SANITARY REVIEW,

AND

### Journal of Public Health.

INCLUDING THE

TRANSACTIONS OF THE EPIDEMIOLOGICAL SOCIETY OF LONDON.

EDITED BY

BENJAMIN W. RICHARDSON, M.D.,

LICENTIATE OF THE ROYAL COLLEGE OF PHYSICIANS,

PHYSICIAN TO THE ROYAL INFIRMARY FOR DISEASES OF THE CHEST, AND LECTURER

ON PUBLIC HYGIENE AT THE GROSVENOR PLACE MEDICAL SCHOOL.

NATIONAL HEALTH IS NATIONAL WEALTH.

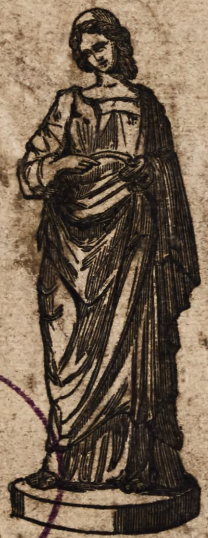

ARMY
MEDICAL
OCT 1 - 1946
LIBRARY

ʽΥΓΙΕΙΑ

*πρεσβίστη μακάρων.*

PRINTED AND PUBLISHED FOR THE PROPRIETOR BY

THOMAS RICHARDS, 37, GREAT QUEEN STREET.

1859.

[Published Quarterly.—Annual Subscription, 10s., Payable in Advance.]

